# Escape performance in the cyclopoid copepod *Oithona davisae*

**DOI:** 10.1038/s41598-024-51288-0

**Published:** 2024-01-11

**Authors:** Marco Uttieri, Leonid Svetlichny

**Affiliations:** 1 Department of Integrative Marine Ecology, Stazione Zoologica Anton Dohrn, Villa Comunale, 80121 Naples, Italy; 2 NBFC, National Biodiversity Future Center, Piazza Marina 61, 90133 Palermo, Italy; 3 Department of Invertebrate Fauna and Systematics, I. I. Schmalhausen Institute of Zoology, National Academy of Sciences of Ukraine, Kyiv, Ukraine

**Keywords:** Ecology, Behavioural ecology

## Abstract

Escaping a predator is one of the keys to success for any living creature. The performance of adults (males, females, and ovigerous females) of the cyclopoid copepod *Oithona davisae* exposed to an electrical stimulus is analysed as a function of temperature by measuring characteristic parameters associated with the escape movement (distance covered, duration of the appendage movement, mean and maximum escape speeds, Reynolds number). In addition, as a proxy for the efficiency of the motion, the Strouhal number was calculated. The escape performance showed temperature-dependent relationships within each adult state, as well as differences between sexes; additionally, changes owing to the presence of the egg sac were recorded in females. In a broader perspective, the results collected reveal the occurrence of different behavioural adaptations in males and females, adding to the comprehension of the mechanisms by which *O. davisae* interacts with its environment and shedding new light on the in situ population dynamics of this species.

## Introduction

The ability of living organisms to interact with their surrounding environment is central to ensure their own survival. Notwithstanding their very small size, planktonic copepods display a diversified repertoire by which adapting their behaviour in response to the stimuli they perceive^1^. These millimetre-long crustaceans sit at the interface between unicellular primary producers and secondary consumers, connecting not only different trophic levels but also the viscous and inertial realms^2,3^, and can act as beacons of climate change^4,5^.

Being prey of a wide gamut of predators from different taxonomic groups and with different feeding strategies, copepods have evolved sensory structures and movement schemes to reduce predation risk^6^. The former include mechanoreceptors sensitive to fluid disturbances created by a nearby hunter^7–9^. The latter are mostly represented by efficient escape responses, fast darting movements performed at speeds of hundreds of mm s^−1^ (or bl s^−1^, where bl represents body length) by which wandering away from the potential threat^6,10^.

All copepod developmental stages are capable of escape responses^11,12^, although the appendages used by juveniles differ from those used by adults^6^. The stage-dependent performance is linked to a stage-dependent predation susceptibility, with increasing escape efficiency during the transition from nauplii to adults^12,13^. In mature copepods, the escape movement begins with a rapid flicking of the first antennae (A1s), which then align along the body to reduce drag^14^, followed by a metachronal front-to-back movement (“kick”) of the swimming legs (pereiopods), starting from the posterior pair, and concluding with a dorsal flipping of the tail (urosome)^15^. Such active propulsive phase continues with the synchronous back-to-front recovery movement of the swimming appendages to their initial position, and the dorso-ventral flipping of the urosome, while the A1s remain pressed against the body^6,14–16^. The stroke and recovery sequence duration typically lasts only a few ms, and it can be repeated at frequencies from tens to hundreds of Hz to ensure a successful escape^14,16,17^. Only when the full escape movement is completed, the A1s stretch back to their open position. The thrust is provided by the sequential movement of the pereiopods^18^ that are spread outward to maximise their surface area^14^, while the movement of the A1 in terms of force generation is still questioned^14,19,20^. The urosome, instead, is primarily used to avoid the strong rotation of the body during the stroke phase as a consequence of the movement of the pereiopods^21^. Numerous factors can influence the escape performance. For example, at low temperatures the latency times and the duration of the kick sequence can be longer^16^, while in turbulent conditions the escape speed may decrease^22^. Interestingly, also the presence of epibionts (as in the case of the cyclopoid *Mesocyclops*^23^) or the consumption of toxin-producing food (based on *Calanus finmarchicus* nauplii^24^ observations) may impair the escape success, thus contributing to a higher predation pressure.

With the aim of further deepening present knowledge on the evasion behaviour of copepods, the temperature-dependent kinematic properties and escape parameters of the cyclopoid *Oithona davisae* Ferrari & Orsi, 1984 are here investigated. Native to the Indo-Pacific area, this species has become a global invader thanks to specific bio-ecological traits favouring its settlement in environments with different ambient conditions^25,26^. In the Black Sea, *O. davisae* was first recorded in 2001^27^, with a population outbreak in Sevastopol Bay in 2005^28^ and subsequent further spreading over the entire basin^29^. A recent field study showed that *O. davisae* population responded positively to the 2010 marine heat wave in the Sevastopol Bay^30^, while experimental trials on both adult females and males from the same site demonstrated the ability of this species to withstand ample salinity changes, females also showing an outstanding osmotic control^26,31^. Sex-specific movement responses were instead reported as a function of temperature: a decrease in temperature to ~ 12 °C determined a reduction in the swimming activity of *O. davisae* males, while females reported a higher degree of adaptability^26^.

In a previous paper^26^, the effect of temperature on the routine swimming behaviour of *O. davisae* was investigated. In the present study, the escape response of adult specimens of *O. davisae* (non ovigerous females, ♀; ovigerous females, ♀_*ov*_; males, ♂) to an external stimulus is studied in relation to environmental temperatures representing typical winter and summer-early autumn conditions in Sevastopol Bay. In particular, this work focuses on the effect of temperature on different parameters used as descriptors of the escape response: distance covered, kick sequence duration, mean and maximum escape speeds, Reynolds number, and Strouhal number. The reactions recorded vary among the sexes, and between the different temperatures and acclimation procedures within each adult state. In addition, differences between ♀ and ♀_*ov*_ are highlighted, cogently owing to the presence of the egg sac impairing the motion of ♀_*ov*_. These outcomes expand current knowledge on *O. davisae* interactions with their environment, complementing previous results focusing on other physiological and behavioural traits^26,31–33^, and improving the comprehension of the mechanisms by which this species can defend itself from an approaching predator. In a wider ecological framework, these results also clarify the population cycle observed in the field, with sex- and stage-dependent patterns of occurrence linked to the differential abilities to cope with varying environmental conditions.

## Results

### Effect of temperature on *Oithona davisae* escape parameters

**Figure 1:**
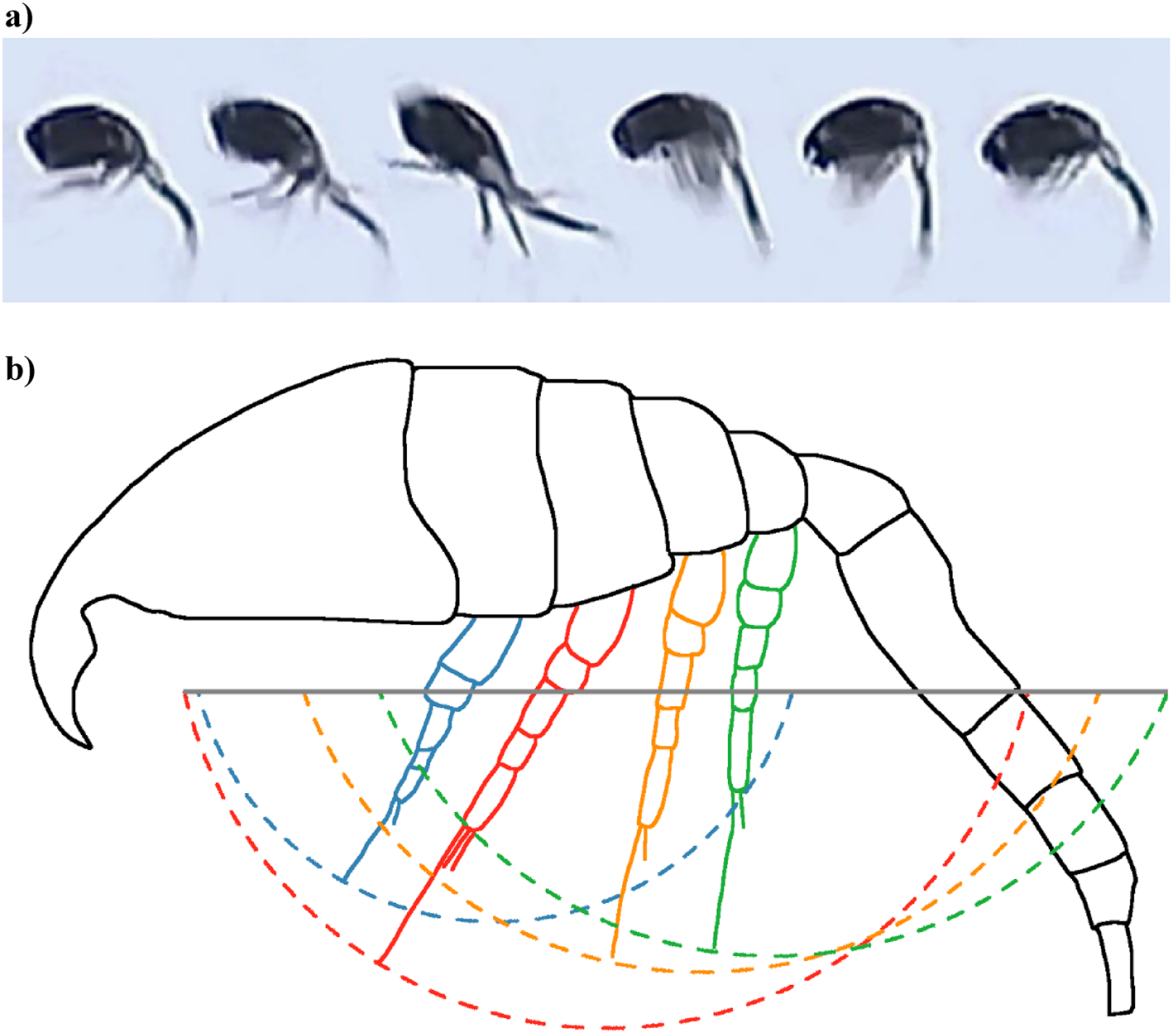
(**a**) Series of single frames extracted from a footage recording the escape jump of an *Oithona davisae* adult female; the sequence shows the metachronal strokes of the pereiopods, followed by the flicking of the urosome and then by the synchronous return movement of all the appendages; (**b**) schematic of the arcs described by the movement of each pereiopod (P1: blue; P2: red; P3: orange; P4: green, from front to rear) of an *Oithona davisae* adult female. The grey line corresponds to the effective peak-to-peak distance L_typ_ covered by the metachronal movement of the pereiopods. *O. davisae* outline redrawn from the original drawings by Ferrari and Orsi ^63^.

The escape reaction of *Oithona davisae* adults (♂, ♀ and ♀_*ov*_) consisted in a series of metachronal front-to-back strokes of the pereiopods (Fig. 1a; Supplementary Video 1) followed by a synchronous back-to-front movement of the swimming legs returning to their initial position. The first kick began with the simultaneous strokes of the antennules (collapsing along the body to reduce drag) and the abdomen (flicking upward/downward), with successive strokes of the four thoracic limbs involved in the motion (starting from P4 and ending with P1) after a short delay, each leg describing an arc during its movement (Fig. 1b). Using a sub-set of video sequences, the average duration of the power stroke by a single pair of legs was estimated as 2.62 ± 0.54 ms. Considering an average total duration of the strokes by the four pereiopod pairs of 7.32 ± 1.89 s at 22 °C^34^, an estimated average delay in the sequence of pereiopod strokes scored as 0.14 times the total duration of the stroke phase.

In all conditions, *O. davisae* adults displayed positive galvanotaxis, with clear responses upon electric stimulation. Due to the unidirectional action of the legs and abdomen, at the beginning of the stroke phase the body axis deviated dorsally. The movement of the body was then levelled off, as the abdomen began a return ventral movement before the swimming legs finished the power strokes. During the recovery phase, the abdomen also struck the ventral side against oncoming water motion, leading to a ventral rotation of the body axis. In the following sections, details on the comparison of the kinematic and escape parameters in the different conditions are presented.

### Influence of acclimation history (22A *vs.* 22W)

**Figure 2:**
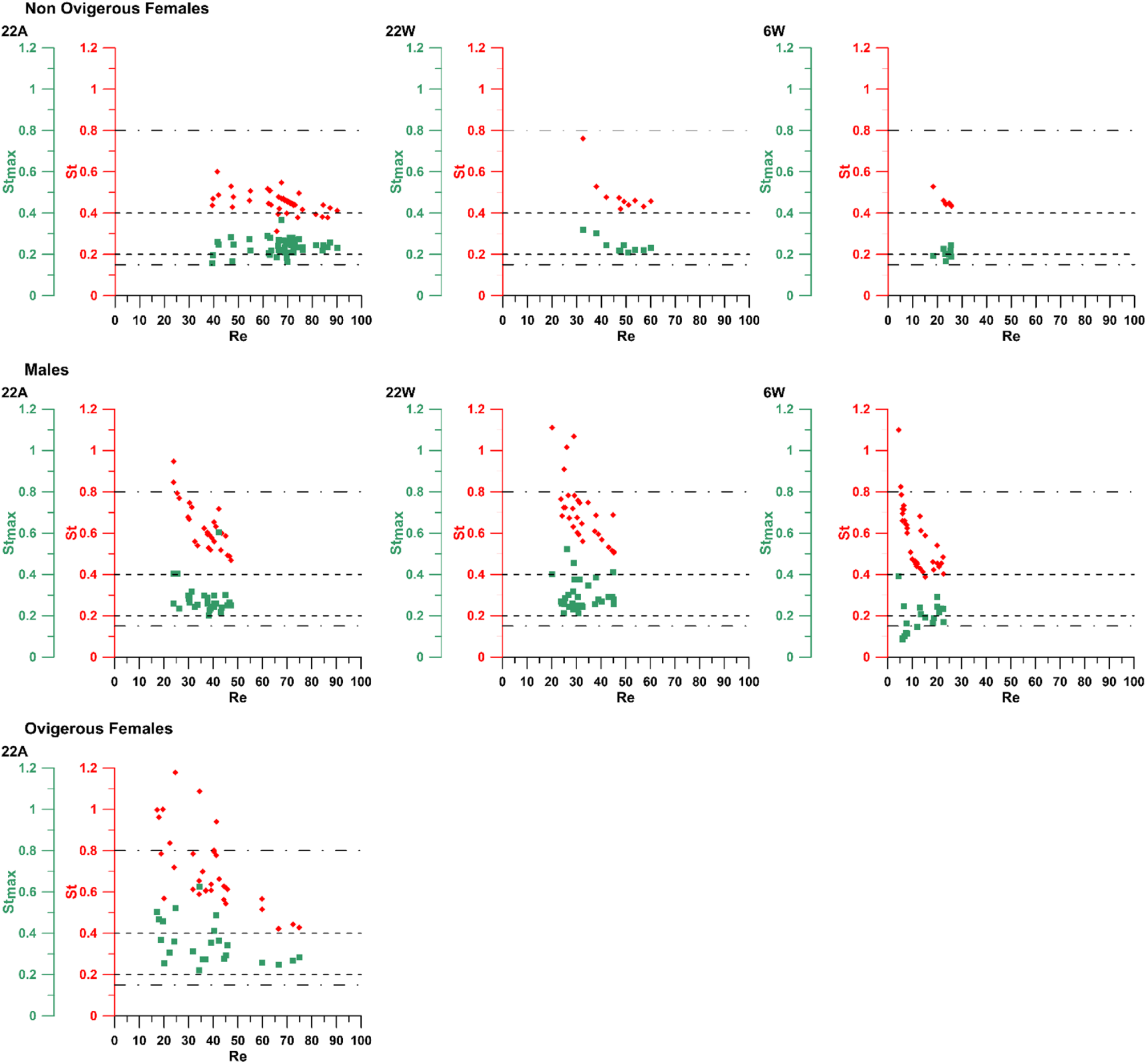
Escape efficiency of *Oithona davisae* adults (♀, ♀_*ov*_ and ♂) in terms of the Strouhal number (St, red) and of the Strouhal number associated with the maximum escape speed (St_max_, green) as a function of Re, for the different experimental conditions tested (22A, 22W and 6W). The dotted lines mark the 0.20–0.40 optimal St range as calculated by Taylor et al. ^53^; the dash-and-dot lines denote the 0.15–0.80 optimal window as resulting from Eloy ^52^.

In ♀ without preacclimation (22A) (Table 1 and Supplementary Table I), the kick sequence duration (t_kick_) was quicker than in preacclimated specimens (22W); no difference was instead recorded in the total distance (Δ) covered during the escape movement. The motion of ♀ at 22A was characterised by higher mean and maximum escape speeds (V_esc_ and V_esc_max_), with associated higher Re; nonetheless, the swimming efficiency in terms of both St and St_max_ was independent of the acclimation history (Fig. 2).

**Table 1:** Summary of the median and interquartile range values of the kinematic and escape parameters used as descriptors of *Oithona davisae* reaction, under the different experimental conditions tested in the work (22A: 22 °C without acclimation; 22W: 22 °C via preacclimation; 6W: 6 °C without acclimation). For comparative purposes with the literature, for each parameter the mean and standard deviation values are reported in square brackets.

	22A			22W		6W	
	♀	♀_ov_	♂	♀	♂	♀	♂
Duration, t_kick_ (ms)	5.83 (0.89) [6.36 ± 1.27]	7.43 (2.25) [7.71 ± 2.22]	6.70 (0.83) [6.81 ± 0.74]	8.30 (1.48) [8.17 ± 0.87]	6.60 (1.70) [7.08 ± 1.07]	11.24 (0.83) [11.35 ± 0.62]	19.16 (9.33) [17.51 ± 4.68]
Distance, Δ (mm)	0.78 (0.07) [0.78 ± 0.09]	0.54 (0.15) [0.56 ± 0.13]	0.55 (0.13) [0.54 ± 0.09]	0.76 (0.06) [0.73 ± 0.11]	0.48 (0.11) [0.49 ± 0.09]	0.79 (0.02) [0.77 ± 0.05]	0.69 (0.20) [0.62 ± 0.14]
Mean escape speed, V_esc_ (mm s^-1^)	131.36 (17.67) [126.84 ± 22.63]	76.19 (23.47) [78.50 ± 30.89]	83.23 (23.33) [79.71 ± 15.11]	91.49 (18.30) [90.37 ± 16.05]	66.47 (23.25) [70.14 ± 15.38]	69.90 (6.50) [68.35 ± 7.14]	37.89 (20.24) [39.98 ± 17.98]
Maximum escape speed V_esc_max_ (mm s^-1^)	242.18 (56.16) [241.70 ± 41.24]	155.33 (62.50) [152.76 ± 49.36]	187.27 (38.00) [180.72 ± 33.91]	184.50 (48.00) [182.10 ± 32.32]	168.00 (33.00) [161.88 ± 23.23]	148.26 (24.22) [152.68 ± 17.36]	140.46 (27.27) [132.92 ± 33.09]
Reynolds number, Re	69.50 (9.35) [67.11 ± 11.97]	40.31 (12.42) [41.54 ± 16.34]	38.17 (10.70) [36.56 ± 69.29]	48.41 (9.68) [47.82 ± 8.49]	30.48 (10.66) [32.17 ± 7.05]	24.17 (2.25) [23.63 ± 2.47]	11.36 (6.07) [11.98 ± 5.39]
Strouhal number, St	0.45 (0.04) [0.45 ± 0.05]	0.63 (0.22) [0.68 ± 0.19]	0.60 (0.16) [0.63 ± 0.12]	0.46 (0.03) [0.49 ± 0.10]	0.68 (0.15) [0.70 ± 0.15]	0.44 (0.01) [0.45 ± 0.03]	0.48 (0.21) [0.56 ± 0.15]
Maximum Strouhal number, St_max_	0.24 (0.05) [0.24 ± 0.04]	0.31 (0.09) [0.34 ± 0.10]	0.26 (0.05) [0.28 ± 0.08]	0.23 (0.03) [0.24 ± 0.04]	0.28 (0.10) [0.30 ± 0.07]	0.20 (0.03) [0.20 ± 0.02]	0.19 (0.07) [0.19 ± 0.08]

Despite t_kick_ and Δ were statistically similar independent of the acclimation process, ♂ escaped faster (both in terms of V_esc_ and V_esc_max_) at 22A, also attaining higher Re and lower St (Fig. 2). No difference was instead recorded in terms of St_max_. Notably, in both conditions ♂ recorded some St values over the 0.80 optimality threshold (Fig. 2).

### Seasonal-dependent escape responses (6W *vs.* 22A)

As a general outcome (Table 1 and Supplementary Table I), the escape response in both sexes was more intense at 22 °C rather than at 6 °C. The kick sequence t_kick_ lasted more than twice in ♀ and more than thrice in ♂; notwithstanding this, Δ was similar in both sexes.

The escape speeds (both V_esc_ and V_esc_max_) were significantly higher at 22 °C, in tandem with the Re reached during the jump. The St in ♀ were statistically similar, while lower St_max_ scores were attained at 6 °C (Fig. 2). In ♂, higher St and St_max_ values were scored at summer-early autumn temperature (Fig. 2). As for the 22A *vs.* 22W case, some St records were higher than the 0.80 upper optimal threshold; in addition, in 6W St_max_ scored also values lower than the lower threshold (0.15) (Fig. 2).

### Sex-specific escape performances (♀* vs.* ♂)

The comparison of the escape performance of *O. davisae* ♀ and ♂ (Table 1 and Supplementary Table I) differed depending on the experimental temperature and acclimation. At 6 °C, ♀ attained shorter t_kick_ and longer Δ than ♂, also achieving almost doubled V_esc_ and associated Re. ♀ also scored lower St, although for both sexes the values were within the optimal window (Fig. 2). No significant statistical difference was recorded between ♀ and ♂ in terms of V_esc_max_ and St_max_.

At 22 °C without preacclimation (22A), the two sexes always behaved differently: ♀ scored shorter t_kick_ and travelled longer distances. V_esc_, V_esc_max_ and Re were also higher in ♀ than in ♂, also scoring lower St and St_max_ (Fig. 2).

When preacclimated to 22 °C (22W), ♀ and ♂ attained statistically similar t_kick_ and V_esc_max_, ♂ reporting smaller Δ. In terms of escape behaviour, ♀ swam faster (V_esc_) attaining higher Re, and lower St and St_max_ (Fig. 2 and Table 1).

### Ovigerous versus non ovigerous females (♀_***ov***_*** vs.*** ♀)

The presence of the egg sac significantly impacted on all the escape parameters analysed. ♀_*ov*_ travelled shorter Δ over longer times t_kick_. In tandem with these differences, the overall escape performance was improved in ♀, which recorded higher mean escape speed V_esc_ at the same stroke phase duration t_kick_ (Supplementary Fig. S1), higher V_esc_max_ and Re, and lower St and St_max_ (Fig. 2). In a number of cases, ♀_*ov*_ also showed St > 0.80, pointing to an under-efficient escape motion (Fig. 2).

The limitations due to the presence of the egg sac was further investigated analysing the dependency of V_esc_ on the total duration of the kick (Supplementary Fig. S1). The regressions clearly indicate that ♀ always scored higher values than ♀_*ov*_, pointing to an impairment on the escape performance owing to the eggs.

## Discussion

An estimated 10,000 species are daily transported across different biogeographic regions on a global scale^35^. Of them, only a very limited fraction becomes established in new environments, formally acquiring the label of non-indigenous species (NIS)^35^. Among the most successful copepod NIS is the cyclopoid *Oithona davisae*^25^, inhabiting eutrophic and transitional waters across different regions worldwide, with a spreading process that is still in course as for example demonstrated by recent records in different areas of the Mediterranean Sea^36–39^. The ability of this species to settle down in receptor environments characterized by different hydrological features is supported by specific physiologic traits as reviewed in Zagami et al.^25^, including a broad salinity range and temperature-dependent behavioural adaptations^26,33,40^. In Sevastopol Bay, this species has also shown a sharp increase in its abundance in response to the summer 2010 marine heat wave^30^, becoming an eligible beacon of the Black Sea warming.

The analysis of the escape behaviour at different temperatures, as investigated in the present study, demonstrates that temperature can differentially affect the behaviour of *O. davisae* adult stages, with possible repercussions in terms of predation susceptibility. Additionally, the results here presented shed new light on the ability of *O. davisae* to cope with and adapt to abiotic factors which may drastically differ from one environment to another, facilitating its establishment and range expansion.

The sequence of pereiopod movement evidenced in this work conforms to previous evidence for the same^34^ and other species^14–16,21^. The incomplete stroke of *O. davisae* P1 during routine jumps reported in Svetlichny et al.^34^ was not evident in the footage analysed here for escape movement. In this case, indeed, the copepod performed full amplitude pereiopod movements, likely ensuring a more hydrodynamically efficient propulsion. Notably, the kinematic scheme of limb movement in the present work was consistent among sexes and experimental conditions.

The experiments presented in this work reveal specific temperature, acclimation and state-dependent escape responses in *O. davisae* upon stimulation. Pre-acclimated (22W) ♀ and ♂ score reduced escape speeds, consequently attaining lower Re, compared to unacclimated specimens (22A), suggesting that the acclimation process may somehow alter copepod performance. This is in line with the acclimation-dependent compensatory thermal shifts in neuromuscular function in other crustaceans, as discussed in Lagerspetz et al.^41^. More investigation is needed to further clarify this issue, in particular by using different acclimation processes as well as different target species.

Wintertime temperatures (6W) inhibit the escapes of the thermophilic *O. davisae* adults. In ♀ and ♂, indeed, the escapes are faster and with quicker appendage movements at 22 °C rather than at 6 °C. These results are convergent with the general temperature-dependent swimming and physiological features of *O. davisae* depicted in previous studies, showing a reduction in motility at low temperatures^26,33,40^. From a physical perspective, the wintertime reduced escape features can be explained considering the higher viscosity of seawater at low temperatures, creating a denser fluid environment which in turn determines slower escape speeds^42^. The decrease in temperature has also physiological implications: it reduces the speed of neural transmission and muscle contraction, as well as that of the appendage movement, as demonstrated in the calanoid *Acartia tonsa* nauplii^43^. However, Lenz et al.^16^ report that in the calanoid *Calanus finmarchicus* the escape parameters show a low dependence on temperature. Indeed, with a difference of 16 °C between the temperatures tested in this work (6W *vs.* 22W), the mean escape speed of *O. davisae* ♀ and ♂ differed by 1.3 and 1.8 times, respectively, which corresponds to temperature coefficients Q_10_ of 1.2 and 1.4, while a temperature Q_10_ coefficient of about 2 is characteristic of changes in the biological rates of aquatic invertebrates, including the routine metabolic rate of *O. davisae*^26^. This may be possibly due to the use of energetic substrates in anaerobic conditions in muscle contraction during escapes^34^.

The comparison of the escape response in ♀ and ♂ points up sex-specific features. In 6W experiments, ♀ escape faster, with larger Δ and shorter t_kick_ than ♂. This outcome goes in tandem with the results discussed above, showing the necessity of ♀ to be responsive even during the winter season. At summer temperature, in absence of any acclimation (22A) ♀ still turn out as being more performing than ♂, while upon thermal adaptation (22W) the two sexes show only minor differences in their behaviour but with overall comparable performances.

Noteworthy, the seasonal-dependent (6W *vs.* 22A) and sex-specific (♀ *vs.* ♂) comparisons provide a complimentary view of the motion behaviour of *O. davisae* adults, at the same time shedding new light on the population-scale strategies of this species. Svetlichny et al.^26^ noted that the salinity tolerance windows of both sexes almost overlap, while their ability to thermo-acclimate differ at low temperatures. In this cyclopoid, ♂ usually swim faster than ♀^26,44^, but when temperatures drop their speed reduces drastically, together with jump frequency and potential distance covered^26^. A similar thermal dependency is here manifested in terms of escape response mechanism, confirming a low-temperature criticality in ♂. Even ♀ abate their locomotory activity and energy metabolism in winter^26,33^, but to a lesser degree compared to ♂. In the Black Sea, the winter population is composed by ♀ only^32,40^, whose survival is ensured by a unique strategy: they are fertilised before the temperature drop, but then delay the production of eggs to spring^33^. As such, for ♀ the ability to maintain active escape mechanisms even at low temperatures, as shown in the present study, is of crucial importance not only at the individual level, but also from a population perspective. Overall, the thermal sensitivity of *O. davisae* behavioural parameters in ♂ is more marked than in ♀, supporting the sex-specific thermal acclimations discussed in Svetlichny et al.^26^.

High-speed (up to 3500 fps) videotaping of escape reaction in *O. davisae* ♀_ov_ and ♀ at 20 °C, taken from continuous cultures, showed that escape jumps were either apparently spontaneous, or provoked by tapping the side of the aquarium or approaching the copepod by a pipette tip^21^. In that report, no statistical difference was scored in the escape parameters between ♀_*ov*_ and ♀^21^, scoring V_esc_max_ and V_esc_ equal to 198 ± 42 and 101 ± 21 mm s^−1^, respectively, covering a distance of 0.58 ± 0.12 mm. Although this last parameter is close to the one reported for ♀_*ov*_ in the present study (Table 1), the mean V_esc_max_ and V_esc_ for egg-bearing *O. davisae* from the current experiments are much lower (152.76 ± 49.36 and 78.50 ± 30.89 mm s^−1^, respectively), while those tallied by ♀ are much higher than those reported in Kiørboe et al.^21^, due to the greater distance of movement during the stroke phase of kick (Table 1). The results point to a clear limitation in the escape mechanism exerted by the presence of the egg sac. In ♀_*ov*_, escapes are slower than in ♀, covering shorter distances over longer times. This evidence supports the recent findings by Svetlichny and Obertegger^45^, reporting slower escape speed in ovigerous females of *Sinodiaptomus sarsi* compared to non ovigerous ones.

**Figure 3:**
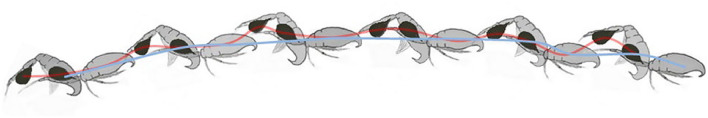
Sketch of the oscillations of *Oithona davisae* ♀_*ov*_ egg sac during six consecutive jumps (left to right). For each jump, two consecutive figures are drawn: the first—leftmost—representing the end of the preparatory phase of the kick (with the abdomen and the egg sac pressed ventrally to the body); the second—rightmost—showing the ♀_*ov*_ during the kick phase with the straightened body. The blue curve shows the trajectory described by the prosome centre, taken as representative of the entire body, while the red one that of the egg sac. The two curves do not overlap, and the oscillations of the egg sac point to a reduced efficiency in the motion.

Figure 3 sketches the trajectories described by the geometric prosome centre (taken as proxy of the entire body) and by the egg sac in six consecutive jumps by one *O. daviase* female, based on original video footage. The two tracks show a clear deviation, and the oscillations of the egg sac likely limit the manoeuvrability of the ♀_*ov*_. The presence of the egg sac thus poses a higher threat on ♀_*ov*_, as their ability to evade from an approaching predator is critically reduced. Indeed, ovigerous copepods have been often reported as more vulnerable to predation compared to non ovigerous ones by both visual and non-visual zooplanktivorous organisms e.g.,^46–48^, representing at the same time an energetically more advantageous food source^49^. The presence of the eggs on females’ urosome may represent a major determinant in predation susceptibility by conferring higher visual conspicuousness^47^, with a positive correlation with clutch size being reported^50^. This would provide a rationale to the evidence that predatory fish score greater attack distances when preying upon ovigerous copepods, independently of their own attack efficiency^49^. On the other hand, the bigger size of ♀_*ov*_ due to the attached eggs would result in a stronger hydrodynamic signal if the individual moved as fast as (or even faster than) ♀, further increasing its predation risk^48^. The reduced swimming activity^49,51^ and escape response (this study) in several egg-bearing copepods may thus be evolutionarily aimed at reducing predation risk and/or result from movement hampering. This however does not seem an universally adopted strategy, as for example demonstrated for the calanoid copepod *Eurytemora affinis*^48^, pointing to possible species-specific strategies.

The peculiar overwintering strategy of *O. davisae* ♀^33^ in the Black Sea can also be interpreted in terms of behavioural advantage compared to ♀_*ov*_. Since the egg sac impairs escape mechanisms, and wintertime ♀ show less intense responses than summertime ones, ♀_*ov*_ at 6 °C would realistically score further reduced reactions than at 22 °C. Fertilised ♀ would thus keep their escape fitness without being limited by the clutch, postponing the production of eggs to more profitable temperature conditions.

Remarkable insights into the efficiency of *O. davisae* escapes are provided by the characterisation in terms of St and St_max_. Independent of the specific comparisons highlighting temperature or sex-related features, the analysis demonstrates that in the majority of cases the three adult stages of the investigated cyclopoid species tally St and St_max_ values within the optimality window predicted by the literature (0.15–0.80)^52^. In particular, for any given condition St_max_ scores values in the lower end of the range (0.20–0.40), corresponding to the most conservative window calculated in Taylor et al.^53^ with a reduced dispersion compared to St, ensuring ideal escapes associated with the fastest movements. The evidence that St and St_max_ tendentially attain values in the predicted most advantageous spectrum indicates that, independent of the kinematic properties of the appendages and the escape features, the evasion movement is nevertheless optimised. In a few cases, however, ♀_*ov*_ and ♂ report St > 0.80, and specifically for ♂, St_max_ < 0.15, *i.e.* values outside the optimal window. This evidence suggests that, out of the three adult states, ♀ are the most performing, while in ♀_*ov*_ and ♂ the escape movement efficiency may be less efficient. Noteworthy, St and St_max_ do not depend on the Re associated with the escape, suggesting a viscosity/inertia independent regulation providing efficiency in any condition.

The St_max_ values for ♀ in the present study align with those reported for *O. davisae*, *Acartia tonsa* and *C. finmarchicus* in^21^. Differently from the present investigation, where the L_ptp_ has been measured considering the arcs described by each pereiopod, Kiørboe et al.^21^ approximated the stroke amplitude to copepods body length. This methodological difference may explain why St_max_, rather than St, converge towards the calculations by Kiørboe et al.^21^. Notwithstanding this aspect, however, both works confirm that the escape motion of *O. davisae* is performed in such a way to optimise the efficiency of the movement.

The use of St in drag-based locomotion, as is the case for metachronal swimming, poses also some concerns with reference to the most appropriate characteristic speed to be used^54^. In the analyses here presented, St and St_max_ have been calculated resorting to the standard forward escape speed of *O. davisae*, as resulting from the motion of the pereiopods. The use of alternative speed proxies, as for example proposed in Murphy et al.^55^, may promote further understanding of the escape efficiency, but this aspect needs additional investigation.

The force generated during copepod escapes is significantly higher than that of other organisms, therefore the movement must be as optimised as possible to result in an energy-effective strategy^21^. The results collected reveal the occurrence of temperature, sex and ovigerous-state dependent behavioural mechanisms in *O. davisae* that may have an impact not only on the fitness of the single individual, but also at the population level. On an evolutionary perspective, the responses of *O. davisae* guarantee an efficient mechanism to flee away from a potential predator, and can be considered an adaptive trait to reduce predation risk and to be favored in newly introduced environments.

## Methods

### *Oithona davisae* sampling and experimental conditions

*Oithona davisae* adult specimens (♂, ♀, and ♀_*ov*_) were collected at the permanent station (depth: 3 m) located opposite the exit of Sevastopol Bay (Black Sea) near the Institute of Biology of the Southern Seas (IBSS) embankment by horizontally towing a 100 µm plankton net (depth: 0.5–1.0 m) at a speed of about 0.5 m s^−1^. The samples were collected in three different periods: ♀ and ♀_*ov*_ in August 2012, at a temperature of 23 °C and a salinity of 17.5; ♂ and ♀ in September 2015 (autumn generation: A), at a salinity of 17–18 and at a temperature of 23–25 °C, and in February 2016 (winter generation: W), at a salinity of 18 and at a temperature of 8 °C. The sampling periods reflected the natural occurrence of the different adult stages in Sevastopol Bay^32^: ♂ and ♀ are more abundant in late summer-autumn, while in winter their abundance decreases sharply; ♀_*ov*_ are absent during the coldest months of the winter-spring period, while the highest clutch size values are recorded in summer. Upon collection, the sample was brought to the laboratory and transferred into a 1 L container. Single adult individuals were pipetted in the vicinity of the illuminated border of the aquarium and transferred into the experimental chambers.

The escape parameters of the three different adult stages of *O. davisae* (see next section) were recorded at different experimental temperatures, or upon different acclimation procedures. Individuals collected in summer 2012 (♀ and ♀_*ov*_) and in autumn 2015 (♂ and ♀) at 23–25 °C were directly transferred to and observed at 22 °C (22A) (sample sizes n: ♀: 50; ♀_*ov*_: 39; ♂: 29). ♀ sampled in winter 2016 at 8 °C were either directly transferred to and observed at 6 °C (6W) (sample sizes n: ♀: 8) or recorded at 22 °C (22W) after gradual preacclimation over 24 h (sample sizes n: ♀: 10). ♂ sampled in winter at 8 °C were either directly transferred to and observed at 6 °C (6W) (sample sizes n: ♂: 38) or gradually acclimated to 14 °C and subsequently transferred to 22 °C until the recording session was initiated (sample sizes n: ♂: 32). Over the course of the acclimation and experiments, the copepods were fed ad libitum on the cryptophyte strain IBSS-CrPr54.

The critical temperature values of 6 °C and 22 °C were selected based on the typical climatology of the Sevastopol Bay, representing harsh wintertime and typical summer-early autumn values, respectively^32,33^. Both summer and autumn ♂ and ♀ were not studied at 6 °C, since they fell into a daze at this temperature.

Depending on the sex and on the experimental conditions, different comparisons were carried out. ♂ and ♀ were separately analysed at 6 and 22 °C (6W *vs.* 22A) to evaluate any seasonal-dependent change in the escape response parameters, and at 22 °C (22A *vs.* 22W) to highlight possible effects of the different acclimation histories on escape fitness. For each temperature and acclimation condition (6W, 22A and 22W), ♂ and ♀ were also contrasted to assess any possible sex-dependent difference. The performances of ♀ (from 22A) and ♀_*ov*_, both collected during warm period, were instead compared to discriminate the potential role of the presence of the egg sac in the escape behaviour. This comparison was carried out only at the highest temperatures tested, in compliance with the seasonal occurrence of ovigerous females, as detailed above.

### Recording of escape behaviour

Five to ten individuals of each adult stage (♂, ♀, and ♀_*ov*_) were separately transferred into 2 mL cuvettes (2.0 × 1.5 × 0.7 cm), and their activity was registered with a Nikon 1 VI (Nikon, Japan) digital camera at a frequency of 1,200 fps, equipped with a long-focus objective (Industar-100U 110 mm, f/4.0, 4 × magnification; USSR) producing a field of view of ~ 12 × 4 mm. Video recordings were performed with a back collimated beam of light produced by a cold (6,000 K) white 5 W LED. After each period of stimulation, the observed individuals were replaced with new specimens. Copepods moving in the focal plane were selected for a frame-by-frame analysis performed using VirtualDub (https://www.virtualdub.org/).

To stimulate the escape reaction, *O. davisae* adults were exposed to a short (5 ms) single electrical impulse (2 Hz) with a current density of 0.05 A cm^−2^ established between silver electrodes located along the opposite walls of the cuvette^56,57^. During the video recordings, the temperature was monitored directly in a chamber with animals to ensure no change in the environment due to the presence of the background filming light.

### Escape kinematic parameters

In order to evaluate the performance of *O. davisae* adults’ behaviour, attention was focused on movement parameters associated with the escape response. In particular, the selected descriptors were: the kick sequence duration (t_kick_, ms), *i.e.* the time taken to complete one full kick cycle (stroke plus recovery phases); the distance (Δ, mm) covered by *O. davisae* individuals during one kick; the mean and maximum escape speeds (V_esc_ and V_esc_max_, mm s^−1^); the Reynolds number (Re) associated with the escape movement, calculated as:

1 $${\text{Re}} = \frac{{{\text{LV}}_{{{\text{esc}}}} }}{{\upnu }}$$

where L was the typical *O. davisae* total body length from Sevastopol Bay (0.518 mm for ♀; 0.449 mm for ♂;^40^), V_esc_ the mean speed during the kick movement, and ν the kinematic viscosity (9.79 × 10^–1^ mm^2^ s^−1^ at T = 22 °C and S = 18; 1.50 × 10^0^ mm^2^ s^−1^ at T = 6 °C and S = 18).

### Strouhal number

During copepod escape, the movement of the pereiopods triggers the formation of impulsive viscous vortex rings in the fluid^18,58^. As an indicator of the escape efficiency, for all conditions the adimensional Strouhal number (St)^52^ was calculated. St corresponds to the ratio of inertial forces due to local acceleration to inertial forces due to convective acceleration, controlling the formation of vortex structures in the wake of a moving body^52^. St is an indicator of the effectiveness of flapping motion^59^, and is given by the formula^52^:

2 $${\text{St}} = \frac{{{\text{fL}}_{{{\text{typ}}}} }}{{\text{U}}}$$

with f representing the beat frequency of the moving appendage, L_typ_ a characteristic length, and U the average forward speed due to thrust. Following the intuitions by^60,61^, St can be calculated also for swimming and flying animals flapping an oscillating airfoil (fin tail, wing) to propel themselves. In such a case, the characteristic length L_typ_ used for the calculation of St typically corresponds to the peak-to-peak distance of the appendage moved to create thrust^52^. In presence of metachronal movement of multiple swimming appendages, the calculation of L_typ_ becomes less straightforward. Considering an organism with one swimming appendage only of length l_sw_, its movement would describe an arc with angle θ whose chord (corresponding to L_typ_) would be equal to:

3 $${\text{L}}_{{{\text{typ}}}} = 2{\text{l}}_{{{\text{sw}}}} \sin \frac{\theta }{2}$$

Such a simplified approach was used by^55,62^ to investigate the efficiency in the motion of the Antarctic krill *Euphasia superba* and of the mantis shrimp *Odontodactylus scyllarus*, respectively. When multiple appendages produce the thrust, the oscillation of each swimming leg may provide additional contributions to the actual L_typ_. In Fig. 1, the arcs described by the each of the four pereiopods involved in the motion of an adult *Oithona davisae* female are sketched (green: P4; orange: P3; red: P2; blue: P1; listed in order of movement, from the posterior to the anterior one). The effective peak-to-peak distance L_ptp_ (solid grey line) originating from the movement of the whole set of pereiopods thus results from the combination of the arcs described by each appendage. In particular, in ♀ the leftmost extremity was covered by P2, while the rightmost one by P4; in ♂, the movement of P1 and P4 contributed to L_ptp_ (figure not shown). It is worth underlining that *O. davisae* possess also a fifth appendage, which is reduced in the females and not contributing to the movement.

The measurement of L_ptp_ in *O. daviase* was performed starting from the original descriptions and drawings by^63^, scaled based on biometric data (prosome length) of adult ♀ and ♂ from Sevastopol Bay^40^, assuming that morphometric ratios did not differ (or at least differed to a non-significant level) among populations. This allowed estimating the pereiopod lengths and the distance between their basal segments. The scaling was performed using GIMP 2.10.30 (freely downloadable at https://www.gimp.org/). Based on Eq. 3 and considering an average angle of movement of the pereiopods $\theta =130^\circ$ (as derived from the visual analysis of the video recordings), the chords defined by the movement of each pereiopod L_P1-4_ were calculated. This $\theta$ value was consistent with those reported for *Calanus finmarchicus*^14^ and *Thermocyclops oithonides*^34^. Considering the L_P1–4_ values, the distance between the basal segments and the pereiopod lengths (Fig. 1), the L_ptp_ for ♀ and ♂ were calculated as equal to 0.35 and 0.33 mm, respectively. These measurements were consistent with a second, independent measurement approach. Considering an intercoxa distance between P4 and P2 of approximately 0.07 mm, we could assume L_ptp_ as the sum of L_P2_ and the distance between P2 and P4 basal segments.

In the case of *O. davisae* escapes, Eq. 2 could then be rewritten as the pereiopods flapped to produce thrust, and St could be calculated as in Eloy et al.^52^:

4 $${\text{St}} = \frac{{{\text{fL}}_{{{\text{ptp}}}} }}{{{\text{V}}_{{{\text{esc}}}} }} = { }\frac{{{\text{L}}_{{{\text{ptp}}}} }}{{{\text{t}}_{{{\text{kick}}}} {\text{V}}_{{{\text{esc}}}} }}$$

where ${\text{f}} = 1/{\text{t}}_{{{\text{kick}}}}$ (s^−1^), and V_esc_ the mean forward speed during the escape (mm s^−1^). Additionally, the St associated with the maximum escape speed was calculated as:

5 $${\text{St}}_{{{\text{max}}}} = \frac{{{\text{L}}_{{{\text{ptp}}}} }}{{{\text{t}}_{{{\text{kick}}}} {\text{V}}_{{{\text{esc}}\_{\text{max}}}} }}$$

For ${\text{St}}>1$, viscosity dominates the flow and the inertial separation of the vortex structures is inhibited, while for small St (O(10^–4^)) a quasi-steady flow arises^64^. At intermediate values, vortex shedding is possible while the flow is not in a steady state^64^. The wakes thus produced can amplify animal net thrust, reducing swimming costs and increasing the optimality of the movement. Triantafyllou et al.^60,61^ calculated optimal St values in the window 0.25–0.35, later expanded to 0.20–0.40 by^53^. Subsequently, Eloy et al.^52^ demonstrated that optimal St values could span from 0.15 to 0.80 for animals encompassing a wide dimensional gamut. It is worth noticing that also other parameters, such as the bending of natural propulsor in different organisms moving in a fluid environment^65^, tend to gather in a predictable range, supporting the adoption of almost-universal mechanisms to ensure highly efficient movement.

### Statistical analysis of escape parameters

The escape parameters for each adult stage and temperature were preliminarily checked for normality using the Shapiro–Wilk test (α = 0.05)^66^, and the normal and detrended QQ plots^67^ (not shown), leading to the assumption of non-normality for each parameter. Dealing with non-normal data, the summary Table 1 reports values as median and interquartile range (IQR); however, for the sake of comparison with other data published in the literature, these values are complemented with mean ± standard deviation. In order to select the most appropriate non-parametric test^68^, for each pairwise comparison the equality of dispersions of the two populations was assessed through the Brown-Forsythe test (α = 0.05)^69^. When homoscedasticity was verified, the Mann–Whitney U test (MW:^70,71^) was selected as test of choice to determine whether the two samples had the same distribution. When the Brown-Forsythe test rejected the null hypothesis, Mood’s median test (MMT:^71,72^) was used to compare the medians of the two samples. For both MW and MMT, a significance level α = 0.05 was used as reference (Supplementary Table I).

### Supplementary Information

Supplementary Information 1.

Supplementary Video 1.

## Data Availability

The data that support the findings of this study are available from the M. U. and L. S. upon reasonable request.
