# Supplementary material for: Escape performance in the cyclopoid copepod Oithona davisae
Source: Sci Rep. 2024 Jan 11;14:1078. doi: 10.1038/s41598-024-51288-0 (PMC10784515; doi:10.1038/s41598-024-51288-0)
Supplement: Supplementary file 1 — Supplementary Information 1. [file 41598_2024_51288_MOESM1_ESM.pdf]

Supplementary Material

Escape performance in the cyclopoid copepod *Oithona davisae*

Marco Uttieri[1,2] & Leonid Svetlichny[3]

[1] Department of Integrative Marine Ecology, Stazione Zoologica Anton Dohrn, Villa Comunale, 80121 Naples, Italy.

[2] NBFC, National Biodiversity Future Center, Piazza Marina 61, 90133 Palermo, Italy.

[3] Department of Invertebrate Fauna and Systematics, I. I. Schmalhausen Institute of Zoology, National Academy of Sciences of Ukraine, Kyiv, Ukraine.

Scientific Reports, https://doi.org/10.1038/s41598-024-51288-0

Supplementary Table I

Summary of the p values for the different pairwise comparisons in the different experimental conditions tested. ** indicates statistically similar results; MW: Mann-Whitney U test; MMT: Mood's median test.

♀ - 22A *vs.* 22W

Parameter	p value	Test
duration, t_{kick} (ms)	5.3×10^{-5}	MW
distance, Δ (mm)	2.7×10^{-1}	MW
mean V_{esc} (mm s^{-1})	1.0×10^{-4}	MW
max V_{esc} (mm s^{-1})	1.6×10^{-4}	MW
Re	1.0×10^{-4}	MW
St	2.7×10^{-1} **	MW
St_{max}	9.2×10^{-1} **	MW

♂ - 22A *vs.* 22W

Parameter	p value	Test
duration, t_{kick} (ms)	6.2×10^{-2} **	MW
distance, Δ (mm)	5.6×10^{-2} **	MW
mean V_{esc} (mm s^{-1})	1.7×10^{-2}	MW
max V_{esc} (mm s^{-1})	6.5×10^{-3}	MW
Re	1.6×10^{-2}	MW
St	4.6×10^{-2}	MW
St_{max}	2.1×10^{-1} **	MW

♀ - 6W *vs.* 22A

Parameter	p value	Test
duration, t_{kick} (ms)	1.0×10^{-9}	MW
distance, Δ (mm)	9.0×10^{-1} **	MW
mean V_{esc} (mm s^{-1})	1.1×10^{-9}	MW
max V_{esc} (mm s^{-1})	2.3×10^{-3}	MMT
Re	2.3×10^{-3}	MMT
St	8.9×10^{-1} **	MW
St_{max}	7.3×10^{-3}	MW

♂ - 6W *vs.* 22A

Parameter	p value	Test
duration, t_{kick} (ms)	8.1×10^{-12}	MW
distance, Δ (mm)	1.1×10^{-1} **	MMT
mean V_{esc} (mm s^{-1})	7.7×10^{-10}	MW
max V_{esc} (mm s^{-1})	1.3×10^{-5}	MW
Re	3.1×10^{-12}	MW
St	6.9×10^{-3}	MW
St_{max}	1.8×10^{-5}	MW

♀ *vs.* ♂- 6 °C

Parameter	p value	Test
duration, t_{kick} (ms)	4.3×10^{-3}	MMT
distance, Δ (mm)	2.0×10^{-2}	MMT
mean V_{esc} (mm s^{-1})	1.9×10^{-3}	MMT
max V_{esc} (mm s^{-1})	1.2×10^{-1} **	MW
Re	2.3×10^{-3}	MMT
St	2.0×10^{-2}	MMT
St_{max}	7.0×10^{-1} **	MMT

♀ *vs.* ♂- 22 °C (22A)

Parameter	p value	Test
duration, t_{kick} (ms)	1.3×10^{-2}	MW
distance, Δ (mm)	6.6×10^{-13}	MW
mean V_{esc} (mm s^{-1})	1.5×10^{-10}	MW
max V_{esc} (mm s^{-1})	2.2×10^{-8}	MW
Re	2.6×10^{-12}	MW
St	5.2×10^{-11}	MMT
St_{max}	9.5×10^{-4}	MW

♀ *vs.* ♂- 22 °C (22W)

Parameter	p value	Test
duration, t_{kick} (ms)	7.4×10^{-2} **	MW
distance, Δ (mm)	3.0×10^{-6}	MW
mean V_{esc} (mm s^{-1})	2.6×10^{-3}	MW
max V_{esc} (mm s^{-1})	7.4×10^{-2} **	MW
Re	7.0×10^{-6}	MW
St	1.9×10^{-5}	MW
St_{max}	4.5×10^{-3}	MW

♀ *vs.* $♀_{ov}$- 22 °C

Parameter	p value	Test
duration, t_{kick} (ms)	2.0×10^{-4}	MMT
distance, Δ (mm)	1.4×10^{-9}	MMT
mean V_{esc} (mm s^{-1})	1.8×10^{-9}	MW
max V_{esc} (mm s^{-1})	7.6×10^{-12}	MW
Re	1.8×10^{-9}	MW
St	1.4×10^{-9}	MMT
St_{max}	5.5×10^{-8}	MMT

Supplementary Figure Captions

Supplementary Figure S1: correlation between mean escape speed V_{esc} with the duration of stroke phase t_{kick} in ovigerous (black circles)and non ovigerous (gray circles) *Oithona davisae* females.

Supplementary Figure S1

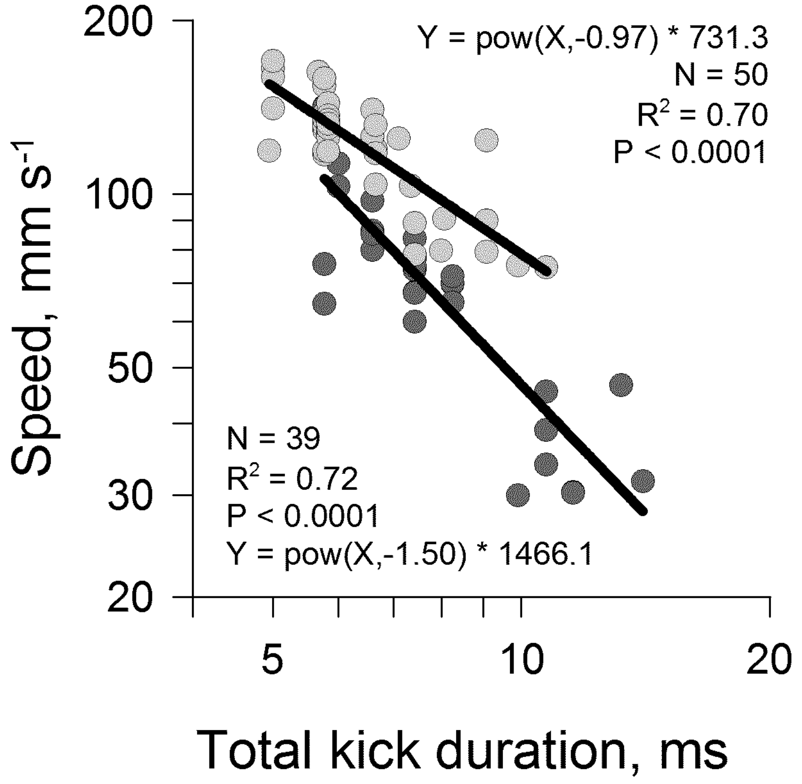

Supplementary Video Captions

Supplementary Video S1: sequence of eight jumps during one escape reaction performed by an ovigerous female of *Oithona davisae*, recorded at 1,200 fps.
